# Impact of Early-Life Environmental Exposures and Potential Transgenerational Influence on the Risk of Coronary Artery Disease and Heart Failure

**DOI:** 10.3390/cells15030222

**Published:** 2026-01-24

**Authors:** Patrycja Obrycka, Julia Soczyńska, Kamila Butyńska, Agnieszka Frątczak, Jędrzej Hałaburdo, Wiktor Gawełczyk, Sławomir Woźniak

**Affiliations:** 1 Student Scientific Society of Heart Diseases, Wroclaw Medical University, 50-556 Wroclaw, Poland; julia.niznik@student.umw.edu.pl (J.S.); kamila.butynska@student.umw.edu.pl (K.B.); agnieszka.fratczak@student.umw.edu.pl (A.F.); jedrzej.halaburdo@student.umw.edu.pl (J.H.); wiktor.gawelczyk@student.umw.edu.pl (W.G.); 2 Division of Anatomy, Department of Human Morphology and Embryology, Wroclaw Medical University, 50-367 Wroclaw, Poland; slawomir.wozniak@umw.edu.pl

**Keywords:** epigenetics, coronary artery disease, heart failure

## Abstract

Cardiovascular diseases (CVDs) remain the leading cause of mortality worldwide and constitute a substantial economic burden. Despite population aging, recent years have witnessed an increasing prevalence of conditions such as heart failure (HF), including among young adults. In this context, coronary artery disease (CAD) has also become an increasingly discussed issue. It has long been recognized that control of risk factors is crucial for prevention. Researchers stress the need to monitor these factors from the earliest stages of life, and detailed analyses indicate an influence of the prenatal period on the development of chronic diseases, including cardiovascular disorders. Transgenerational and intergenerational epigenetic mechanisms are also taken into account. This review aims to systematically evaluate the existing literature and summarize the mechanisms that may link these factors. We consider epigenetic, metabolic, immunological, and inflammatory influences. We describe examples of environmental exposures, such as air pollution, maternal diet, toxins, and infections, and analyze data derived from clinical studies. We discuss gaps in the literature and identify limitations, outlining directions for future research and emphasizing the need for CVD prevention initiated at the earliest stages of life.

## 1. Introduction

According to the World Health Organization (WHO), cardiovascular diseases (CVDs) are the leading cause of death worldwide, accounting for 32% of all global deaths in 2022 (19.8 million). Data from this overview indicate that myocardial infarction (MI) and stroke dominate as causal factors, while geographically the greatest burden is borne by low- and middle-income countries [1]. Furthermore, ischemic heart disease remains the leading contributor to global mortality and morbidity, being responsible for 8.99 million deaths in 2021 [2], whereas heart failure (HF) has reached pandemic proportions, currently affecting an estimated 64 million people worldwide [3]. CVDs continue to represent a substantial economic burden, generating both direct healthcare expenditures and indirect costs related to productivity loss and premature mortality. Statistics from the United States indicate that in 2019, CVDs and stroke generated USD 251 billion in direct costs and USD 156 billion in indirect costs. Significant consequences are also observed in populations younger than 65 years of age [4]. Europe likewise faces major challenges. In 2021, total costs reached EUR 282 billion, of which EUR 155 billion was attributed to healthcare and social care systems. Productivity losses were estimated at EUR 48 billion [5]. The increase in CVD incidence since 1990 has been attributed, among other factors, to population aging [6]. However, in the case of HF, a rising trend has been observed in recent years among young adults, and an increase in its prevalence among individuals aged 15–49 years is also projected over the coming decade [7,8]. Coronary artery disease (CAD) is likewise increasingly discussed in the context of young adults, with its frequency in this group described as stable or rising [9]. According to the WHO, control of risk factors is of key importance in the prevention of CVDs [1]. The literature divides these factors into classical—on which clinical practice primarily focuses—and non-classical risk factors. The former include, among others, arterial hypertension and diabetes mellitus, whereas the latter encompass gut microbiota, diet, and sleep [10]. Studies emphasize the necessity of already controlling these factors in early life in order to prevent CVDs later in life [11]. A detailed analysis of the origins of chronic diseases, including cardiovascular disorders, must take into account not only postnatal but also prenatal influences [12]. It is well-established that from conception to the postnatal period, the fetus is exceptionally sensitive to environmental fluctuations due to complex developmental processes, and even minor disturbances may alter anticipated developmental trajectories [13]. According to the Barker hypothesis, the development of certain adult diseases is associated with intrauterine environmental influences and fetal adaptive responses [12]. The origins of this concept stem from observations of increased mortality due to ischemic heart disease among individuals with low birth weight [14]. For example, intrauterine growth restriction, occurring in approximately 10% of pregnancies and often resulting from maternal factors, exerts long-term health consequences in offspring, including cardiovascular complications [15]. These considerations are linked to the concept of the Developmental Origins of Health and Disease (DOHaD), which assumes that disease risk in adult life is shaped from the moment of conception [14]. This perspective has transformed the understanding of many diseases by expanding classical paradigms. Authors now emphasize the need to investigate intrauterine influences alongside heredity and lifestyle in the search for new insights [16]. Epigenetics plays a central role in this context. In the literature, regulatory mechanisms such as DNA methylation and histone modifications are cited as examples, both of which are influenced by numerous environmental factors that ultimately alter CVD risk [17]. In addition to intrauterine growth restriction, preeclampsia and gestational diabetes may also exert profound fetal effects through epigenetic mechanisms. These consequences include altered expression of genes related to vascular function and atherosclerosis-associated pathways [18]. Of particular importance is transgenerational epigenetic inheritance, defined as the persistence of epigenetic modifications across multiple generations without changes in the DNA sequence. In such cases, generations that were not directly exposed to the environmental factor are also affected [19,20]. The literature highlights potentially serious implications, including epigenetically mediated susceptibility to obesity and diabetes in subsequent generations [21]. Transgenerational inheritance should be distinguished from intergenerational inheritance, in which parental exposure results in consequences for the immediate offspring. Studies have analyzed the impact of maternal intake of nutrients or various chemical substances on health outcomes in future generations [19], and paternal dietary influences have also been investigated [22]. Notably, reports indicate that exposure to air pollution and heavy metals during fetal development may contribute to congenital heart defects. From a public health perspective, such findings may potentially necessitate a reassessment of environmental protection strategies, although authors emphasize the need for further research [23]. In relation to climate change—considering projections of a 1.5 °C increase in mean annual global surface temperature by 2050—it has been confirmed that maternal hyperthermia affects the development of congenital heart defects, likely through mechanisms involving heat shock proteins [24]. Authors also emphasize the roles of oxidative stress and gut microbiota dysregulation within the DOHaD framework. They report potential utility of antioxidants, such as vitamins and amino acids, for which preclinical studies have demonstrated beneficial effects, suggesting the possibility of reducing CVD risk in offspring [25]. Long-term morbidity is also discussed as a consequence of conditions such as preterm birth, and prenatal care is highlighted as a key element in preventing adverse outcomes. This includes medical examinations, screening for potential infections, dietary counseling, and psychosocial interventions aimed at supporting emotional regulation and thereby limiting cortisol exposure [26].

**Figure 1:**
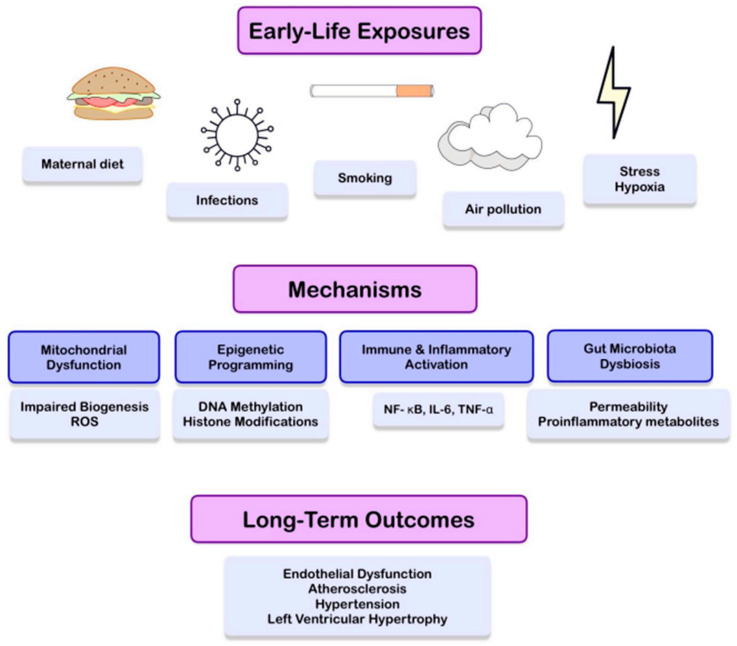
Conceptual overview of developmental programming of cardiovascular disease risk. The figure illustrates early-life environmental exposures (including maternal diet, infections, smoking, air pollution, and psychosocial or hypoxic stress) and their influence on key mediating mechanisms such as mitochondrial dysfunction, epigenetic programming, immune and inflammatory activation, and gut microbiota dysbiosis. These interconnected pathways may lead to long-term cardiovascular outcomes, including endothelial dysfunction, atherosclerosis, hypertension, and left ventricular hypertrophy.

A preliminary review of the existing evidence suggests a potential role of early environmental exposures in shaping CVD risk. We attempt to systematize the data published in this area. We summarize mechanisms that may link early exposures to changes in the heart and vasculature, including epigenetic, metabolic, immunological, and inflammatory mechanisms, drawing attention to their potential coexistence and interactions. We also address transgenerational phenomena. We describe examples of environmental exposures—such as air pollution, maternal diet, toxins, and infections—and discuss evidence derived from clinical studies. Finally, we analyze existing gaps in the literature and identify potential limitations. We attempt to outline directions for future research, emphasizing the necessity of preventing CVDs beginning at the earliest stages of life. Figure 1 provides an overview of a conceptual pathway linking early-life environmental exposures with key mediating mechanisms (including epigenetic, metabolic, immunological, inflammatory, and microbiota-related processes) and their long-term cardiovascular consequences.

## 2. Materials and Methods

This narrative review is informed by a non-systematic search of PubMed, Google Scholar and Web of Science databases conducted between 14 November 2025, and 15 January 2026, focusing mainly on human observational and interventional studies from the past 15 years, while allowing inclusion of earlier landmark studies dating back to 2000 where relevant. Representative search terms included combinations of “early-life exposures,” “developmental programming,” “cardiovascular disease,” “epigenetics,” “DOHaD,” “air pollution,” “maternal diet,” “gut microbiota,” and “heart failure.” The literature search was designed to capture key epidemiological evidence addressing early-life environmental exposures and long-term cardiovascular outcomes. Study selection was guided by relevance to prenatal, early-life, intergenerational and transgenerational risk factors for cardiovascular disease. Studies were screened by title and abstract, followed by a full-text review of the potentially relevant article. All figures were created by the authors using GoodNotes (version 7.0.26; GoodNotes Limited, London, UK).

## 3. Mechanistic Pathways Linking Early-Life Exposures to CAD and HF Risk

Early life—including the prenatal period and early childhood—is a sensitive window for cardiovascular system development. Evidence from both epidemiological observations and experimental models indicates that environmental factors during this period may exert lasting effects. The DOHaD hypothesis suggests that prenatal or postnatal stress may program the risk of cardiovascular disorders. Animal and human studies demonstrate that developmental exposures such as undernutrition, toxins, stress, pollutants, or medications are consequential [27,28]. The effects of these factors are mediated through epigenetic, metabolic, immunological, and mitochondrial alterations, as well as through the modulation of the microbiome. These changes are hypothesized to result in persistent modifications of vascular and cardiac structure and function, thereby increasing long-term risk of CAD and HF [29].

### 3.1. Epigenetic Mechanisms

Epigenetic mechanisms, including DNA methylation, histone modifications, and miRNA-mediated regulation, play a critical role in stabilizing the effects of the prenatal environment [30]. Studies in animal models have demonstrated that maternal nutritional deficiencies lead to persistent epigenetic disruptions in offspring tissues. A low-protein diet induces alterations in the methylation of promoters of genes essential for metabolism and endocrine cell function. Even short-term prenatal exposure appears to be sufficient to program long-term phenotypes that predispose individuals to metabolic disorders and CVDs [31,32]. These effects also include intergenerational consequences. While animal models provide robust evidence for these pathways, it is important to note that direct causal evidence in humans is more challenging to establish due to confounding environmental factors. Protein restriction during pregnancy modifies DNA methylation profiles in offspring adipose tissue. Some of these changes persist across subsequent generations in rodent models, indicating the potential transmission of epigenetic effects of protein undernutrition. Additionally, a low-protein diet affects the expression of epigenetic enzymes, such as DNA demethylases, thereby reinforcing aberrant methylation of PPARα and other genes regulating metabolism [33]. In the cardiovascular context, this leads to metabolic reprogramming of the heart, increased susceptibility to hypertrophy, mitochondrial dysfunction, and excessive production of reactive oxygen species (ROS), thereby accelerating the development of cardiac pathologies in adulthood [34].

### 3.2. Mitochondrial Mechanisms

Additionally, a growing body of evidence indicates that prenatal stress can induce persistent impairments in mitochondrial function, representing another mechanism underlying cardiovascular risk programming. Fetal exposure to stressors—such as inflammation, hypoxia, or excess glucocorticoids—leads to impaired mitochondrial biogenesis, increased ROS production, and disruptions in oxidative phosphorylation, thereby promoting the persistence of a proinflammatory and proatherogenic metabolic phenotype in tissues [35,36]. Experimental studies on animals have shown that prenatal hypoxia induces long-lasting mitochondrial dysfunction in the offspring’s heart, increasing susceptibility to endothelial dysfunction, left ventricular hypertrophy, and the development of cardiovascular pathologies, thereby supporting the role of mitochondrial mechanisms in programming the risk of CAD and HF [37].

### 3.3. Nutritional Deficiencies and Metabolism

Similar consequences are observed with deficiencies in B vitamins, particularly vitamin B12 and folate, which serve as key methyl group donors. Even during the periconceptional period, inadequate intake of these nutrients disrupts gene expression and methylation patterns associated with the renin–angiotensin system, phospholipid homeostasis, and mitochondrial function. In adulthood, these alterations contribute to insulin resistance, hypertension, and increased susceptibility to CVDs [38]. Fetal growth restriction also induces epigenetic changes in genes related to vascular function, particularly in regions controlling the expression of nitric oxide synthase (NOS). This results in reduced nitric oxide (NO) production and persistent endothelial dysfunction in various experimental settings [39]. Moreover, NO deficiency, a key mediator of vascular relaxation, promotes increased vascular resistance and the development of hypertension [40]. It has also been demonstrated that stable differences in DNA methylation present at birth in individuals with very low birth weight predict later adverse cardiovascular phenotypes, reflecting early programming of susceptibility to CAD and HF. These mechanisms indicate that environmental disturbances in early life can initiate long-lasting, and potentially even transgenerational changes in vascular and metabolic regulation, thereby increasing the risk of CVDs in adulthood [41]. However, it should be noted that some longitudinal studies have reported inconsistent results regarding the stability of these methylation markers over the lifespan, suggesting that postnatal environment may partially mitigate or exacerbate initial programming.

### 3.4. Renin–Angiotensin–Aldosterone System and Histone Regulation

Another key component of epigenetic programming involves alterations in the function of the renin–angiotensin–aldosterone system. Angiotensinogen, a protein produced primarily in the liver and a precursor of angiotensin I, which—under the action of renin and angiotensin-converting enzyme—regulates blood pressure, fluid balance, and local cardiovascular processes including inflammation, cell proliferation, and migration in both the systemic circulation and tissues, is also subject to epigenetic regulation [42]. Consequently, demethylation of the Angiotensinogen gene promoter increases its expression in tissues, leading to higher levels of angiotensin I and II, suggesting a mechanism that may contribute to elevated blood pressure and increased risk of hypertension [43]. Histone H3/H4 modifications and the enzymes acting on them, namely histone acetyltransferases and histone deacetylases (HAT/HDAC), regulate the expression of inflammatory and proatherogenic genes in vascular wall cells, thereby promoting the development of atherosclerosis [44]. Moreover, studies in preclinical models have shown that HDAC inhibition improves cardiac function by increasing myofilament calcium sensitivity and reducing diastolic tension, which limits the development of cardiomyocyte hypertrophy and enhances myocardial contractility [45].

### 3.5. Role of miRNA

miRNAs also mediate developmental programming: maternal diet alters the offspring miRNA profile, including miRNAs that regulate lipid metabolism, insulin sensitivity, and inflammatory processes [46]. One such subgroup comprises inflamma-miRs, including miR-21, miR-155, and miR-146a, which modulate oxidative stress and inflammatory responses in cardiac muscle [47]. In patients with CAD, significant alterations in miR-155 expression have been observed, indicating its role in regulating inflammatory processes and oxidative stress in the pathogenesis of CAD. Changes in the expression of other inflammation-related miRNAs further suggest that circulating inflamma-miR profiles may serve as noninvasive biomarkers of inflammatory status and cardiovascular risk [48]. Furthermore, emerging evidence suggests that measurement of specific miRNAs in umbilical cord blood or early neonatal samples may have utility as early biomarkers of cardiovascular risk phenotypes, including structural heart defects and later-life cardiovascular risk factors. For example, certain miRNAs in cord blood have demonstrated diagnostic performance for congenital heart disease (e.g., AUC up to 0.86), and postnatal miRNA profiles have been linked to early cardiovascular risk indicators in childhood. However, longitudinal evidence directly linking neonatal miRNA levels to later adult CAD or HF outcomes remains limited and warrants further prospective research [49].

### 3.6. Immune System

Another key aspect of early cardiovascular programming is activation of the immune system [50]. Of particular importance is stimulation of the fetal NF-κB pathway in response to maternal inflammation or infection. This leads to increased levels of IL-6 and TNF-α, which program chronic vascular inflammation. Studies in rats have shown that prenatal exposure to lipopolysaccharide results in persistent activation of NF-κB in the offspring’s aorta, inducing hyperreactivity of the renin–angiotensin–aldosterone system, increased IL-6 and TNF-α levels, and the development of hypertension [51].

### 3.7. Early Development of the Gut Microbiota and Its Significance

**Figure 2:**
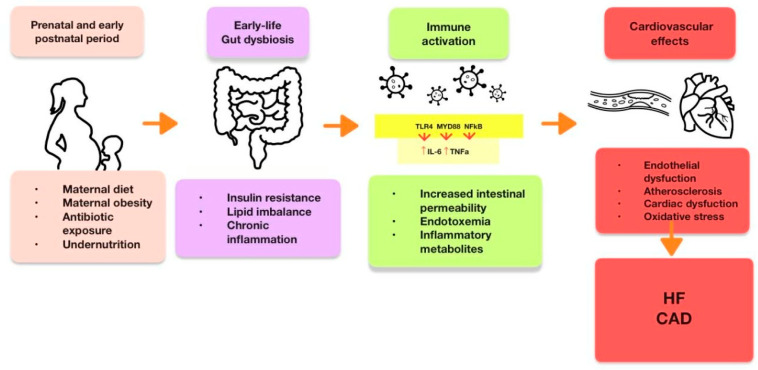
The gut–heart axis in early-life programming. Dysbiosis disrupts the gut–immune axis, activating proinflammatory pathways and increasing cardiovascular disease risk through increased intestinal permeability and microbial metabolite production.

In parallel, early development of the gut microbiota plays a crucial role in regulating inflammation and host metabolism. Gut dysbiosis during the prenatal or early postnatal period may disrupt the maturation of the immune and metabolic systems. Literature reviews indicate that alterations in the microbiota during this critical window are associated with an increased risk of obesity, insulin resistance, and CVDs [52,53]. A growing body of evidence also demonstrates that environmental conditions during the prenatal and early infancy periods—such as maternal diet, obesity, undernutrition, or exposure to antibiotics—can induce long-lasting changes in the offspring’s microbiota composition. These alterations affect body weight regulation, insulin sensitivity, lipid metabolism, and the development of low-grade chronic inflammation, which constitutes a significant risk factor for cardiovascular disorders later in life [54]. Dysbiosis during a critical developmental period also promotes disruption of the gut–immune axis. Increased intestinal permeability, enhanced endotoxemia, and the production of proinflammatory metabolites activate the TLR4/MYD88/NF-κB signaling pathways, promoting elevated levels of proinflammatory cytokines, including IL-6 and TNF-α. These mechanisms directly affect the endothelium, accelerating atherosclerotic processes and modulating cardiac muscle function [55]. Furthermore, trimethylamine N-oxide (TMAO), a metabolite produced by gut microbiota from dietary precursors such as choline, L-carnitine, and betaine, is increasingly recognized as a mediator of cardiovascular risk [56]. Elevated TMAO levels in mothers or newborns have been associated with endothelial dysfunction, increased oxidative stress, proinflammatory signaling, and increased susceptibility to atherosclerosis and heart failure. Recent studies suggest that TMAO may serve not only as a biomarker but also as a potential target for early preventive interventions [57]. However, although microbiota-directed strategies such as fecal microbiota transplantation have shown promising effects in restoring vascular health in animal models [58], their application in human developmental programming remains speculative and requires clinical validation. Figure 2 presents the role of the gut–heart axis in early programming.

### 3.8. Transgenerational Phenomena

An increasing number of studies also describe transgenerational phenomena. Transgeneration refers to the transmission of metabolic susceptibilities to offspring not only through genetic inheritance but also via epigenetic modifications arising in response to factors acting before conception or during the perinatal period [59]. These changes may originate in parental germ cells or result from fetal exposure to an adverse intrauterine environment, leading to persistent epigenetic reprogramming and an increased risk of metabolic disorders in subsequent generations [60]. Animal models have demonstrated that parental factors can modify the epigenome of germ cells, as evidenced by studies showing that traumatic stress in young male mice alters the sperm miRNA profile, resulting in metabolic disturbances in the offspring [61]. In humans, the available data are limited and direct transgenerational epigenetic inheritance remains a subject of active scientific debate. However, the well-known Överkalix studies demonstrated that the nutritional conditions of grandparents influenced the health of grandchildren. Food scarcity experienced by great-grandfathers was associated with an increased risk of diabetes and all-cause mortality in grandchildren, whereas adequate food availability reduced this risk [62]. No association was found between grandparents’ diets and CVDs in grandchildren. These findings suggest that, during specific developmental windows, epigenetic signals (e.g., DNA imprinting or miRNAs) may transmit information across generations; however, the lack of consistent findings across different human cohorts and the complexity of socioeconomic factors mean that these mechanisms require much more cautious interpretation and further investigation [63].

### 3.9. Summary

In light of the presented evidence, early life represents a critical window of vulnerability during which environmental factors initiate persistent, epigenetically stabilized alterations involving metabolism, vascular function, the immune system, mitochondria, and the gut microbiota, thereby increasing the risk of CAD and HF in adulthood. This process can be conceptualized through a unifying theoretical framework where environmental exposures act as “first hits,” triggering a cascade of epigenetic modifications that alter gene networks (e.g., NF-κB, RAAS, and metabolic pathways). These molecular changes converge on systemic phenotypes characterized by chronic inflammation and mitochondrial dysfunction, which ultimately dictate the adult disease trajectory. A growing body of evidence also indicates that some of these alterations may persist across generations, underscoring the importance of the early-life environment as a key determinant of cardiovascular health in subsequent generations. From a precision prevention perspective, early risk stratification may be informed by integrative biomarker panels, including cord blood DNA methylation patterns, maternal or neonatal gut-derived metabolites (e.g., TMAO precursors), and selected circulating miRNAs, enabling targeted nutritional or lifestyle interventions before or during pregnancy. Table 1 presents a summary of the key findings.

## 4. Examples of Environmental Exposures

### 4.1. Air Pollution

**Figure 3:**
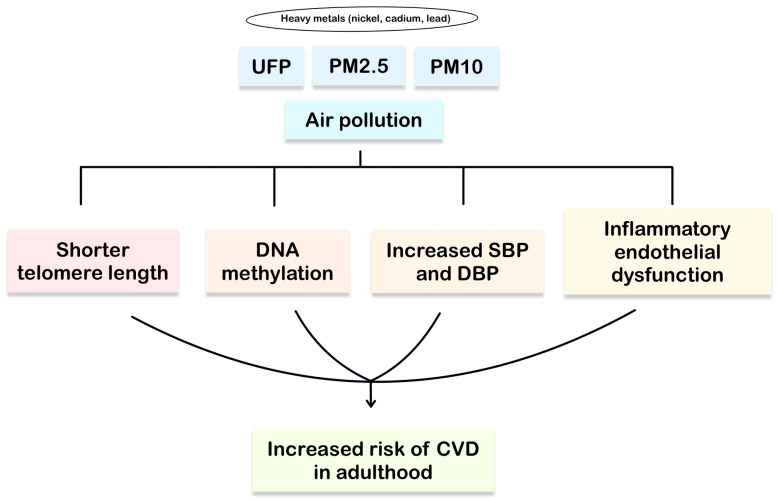
Air pollution exposure and cardiovascular disease development. Early-life air pollution exposure triggers oxidative stress and systemic inflammation, programming persistent vascular dysfunction and increased atherosclerosis risk.

According to the WHO, air pollution is responsible for approximately 6.7 million deaths worldwide each year. The vast majority of these deaths are attributable to conditions such as CVDs, lung cancer, chronic respiratory diseases, and stroke [64,65]. Air pollution is defined as a mixture of gases and particles whose origin, composition, and toxicity vary temporally and spatially [66]. Particulate air pollutants can be classified according to their size. They are categorized as particles with a diameter of less than 0.1 μm (ultrafine particles, UFP), less than 2.5 μm (PM_2.5_), and less than 10 μm (PM_10_) [67]. In a study by Zhang M et al., exposure of pregnant women during the third trimester to PM_2.5_ was shown to be associated with increased blood pressure in children aged 3–9 years. Moreover, PM_2.5_ exposure may be associated with shorter telomere length in children, which in turn may increase the risk of CVDs in adulthood [68]. Prenatal exposure to air pollution may lead to DNA methylation of repetitive elements such as Alu and LINE1, which may affect genes regulating cardiovascular development. Studies by Breton et al. also demonstrated that prenatal exposure to NO_2_ during the third trimester is potentially associated with higher blood pressure in 11-year-old children [69]. In contrast, the Project Viva study showed that increased neonatal systolic blood pressure (SBP) and diastolic blood pressure (DBP) may be associated with greater exposure to PM_2.5_ during the 2–7 days preceding delivery. Nickel alone may potentially increase SBP and DBP, whereas zinc may reduce them. Furthermore, nickel is thought to be involved in leukocyte recruitment to blood vessels, ultimately leading to inflammatory endothelial dysfunction [70]. Lead, a heavy metal, may also exert adverse effects on CVDs. Population-based studies in adults suggest its negative impact on blood pressure [24]. In the prospective cohort study, Strong Heart Study, urinary cadmium levels were analyzed. High levels of this metal were found to be associated with an increased incidence of CVD- and CVD-related mortality. Additionally, cadmium exposure was linked to subclinical cardiac damage and MI [71]. Figure 3 illustrates how air pollution contributes to an increased risk of developing CVDs.

### 4.2. Influence of Maternal Diet

Maternal diet plays a critical role in the proper development of the fetal cardiovascular system. Inadequate nutrition during pregnancy may be associated with an increased risk of CAD, stroke, and hypertension in the offspring during adulthood [72]. Moreover, it has been demonstrated that nutritional interventions during the first 1000 days of life (from conception to 2 years of age) may yield greater health benefits than the treatment of noncommunicable diseases in adulthood [73]. Studies by Ferey et al. showed that maternal obesity induced by a high-fat, high-sugar diet can lead to persistent intergenerational alterations in cardiac structure and function in offspring. Mitochondrial cardiac defects were identified in both male and female mice, which may be linked to nuclear epigenetic modifications. Importantly, subsequent generations of offspring may also be exposed, either directly or indirectly, to the effects of maternal obesity [74]. Other murine studies indicate that offspring of obese mothers may exhibit re-expression of cardiac genes (including ACTA1, MYH6/MYH7, and NPPB), which promotes the development of pathological cardiac hypertrophy. Additionally, systolic and diastolic dysfunction, along with reduced expression of troponin I and SERCA2a—proteins essential for normal myocardial contractility—have been observed. These changes may predispose individuals to premature HF [75]. Not only maternal obesity but also insufficient protein intake throughout pregnancy adversely affects offspring health. A low-protein diet leads to reduced expression of the 11β-HSD2 gene, resulting in increased activity of the hypothalamic–pituitary–adrenal axis and potentially contributing to the development of arterial hypertension in rat offspring [76]. Excessive sugar intake during pregnancy likewise exerts a significant impact on the offspring’s cardiovascular system. Reducing maternal dietary sugar may enhance protection against future cardiovascular problems [73]. Animal studies have shown that maternal diabetes can induce DNA methylation and histone modifications, thereby affecting genes that regulate cardiac function. Children of mothers with diabetes more frequently exhibit left ventricular hypertrophy, higher blood pressure, and increased vascular stiffness, which may promote the development of heart disease [77]. Furthermore, excessive fructose intake during pregnancy may contribute to the development of preeclampsia, increasing the risk of stroke in the offspring during adulthood [78]. In contrast, evidence regarding the effects of maternal intake of n-3 (ω-3) fatty acids on vascular stiffness and blood pressure in offspring remains inconsistent. Some studies suggest beneficial effects, such as reduced vascular stiffness, whereas others report no significant impact [79].

### 4.3. Smoking During Pregnancy

In 2018, published data estimated that smoking during pregnancy occurred in approximately 1.7% of women worldwide [80]. Smoking during pregnancy may predispose offspring to an increased risk of MI and stroke later in life [81]. Moreover, a parental history of smoking may also be associated with an elevated risk of CVDs in offspring, including atrial fibrillation, MI, and HF. Parental smoking status may additionally be related to the smoking behavior of offspring [82]. Cigarettes contain harmful substances such as nicotine, carbon monoxide, and ROS, which can disrupt the structure and normal functioning of the cardiovascular system [81]. Nicotine can cross the placenta into the amniotic fluid and fetal circulation and bind to nicotinic acetylcholine receptors, resulting in vasoconstriction and reduced placental blood flow. This leads to elevated blood pressure and cardiovascular problems in offspring during adulthood [80,81]. In a cohort study conducted by Wang T et al., active maternal smoking within three months prior to pregnancy was shown to increase the risk of congenital heart disease (CHD) in offspring by 165%, while passive smoking increased the risk by 69% [83]. This aspect is particularly important given the substantially increased risk of HF among individuals with CHD compared with the general population. In the study by Gilljam et al., the risk of developing HF was reported to be more than 100-fold higher in patients with CHD than in individuals without CHD. Moreover, patients with more complex congenital heart defects exhibited an even greater predisposition to the development of HF [84].

### 4.4. Oxidative Stress as a Common Mechanism of Environmental Exposures

Oxidative stress is defined as an imbalance between increased levels of ROS and reduced activity of antioxidant defense mechanisms [85]. It may lead to abnormal fetal development and the occurrence of CVDs. It also predisposes one to cardiac hypoxia, which in turn may be associated with myocardial hypertrophy, increased expression of NADPH oxidase, and the development of hypertension [86]. Increased methylation of the NR3C1 gene at CpG (cytosine–phosphate–guanine) sites leads to reduced gene expression, thereby disrupting negative feedback regulation of the hypothalamic–pituitary–adrenal axis. As a result, cortisol levels increase, which may predispose individuals to cardiovascular disorders later in life [87].

### 4.5. Infections

In a meta-analysis of 17 case–control studies, the prevalence of offspring with CHD was assessed among children born to mothers infected with a virus during early pregnancy compared with those without such infection. The analysis showed that maternal viral infection was associated with an increased risk of giving birth to a child with CHD. This association was strongest for rubella virus and cytomegalovirus. However, the authors emphasized substantial heterogeneity among the included studies, indicating that this topic requires further investigation [88,89]. In a PROSPERO-registered systematic review and meta-analysis, it was demonstrated that among the most common congenital heart defects associated with infections were ventricular septal defects and atrioventricular septal defects. Despite several viral infections that may potentially influence the occurrence of CHD in offspring, no such associations were identified for bacterial infections [90]. Currently, there is limited scientific evidence describing the transgenerational impact of infections on the development of heart failure. One hypothesis proposes that viral infection may potentially influence the expression of genes associated with cardiomyopathy [91].

## 5. Clinical Evidence

Integrating data from clinical and population-based studies, the following section discusses key evidence supporting the hypothesis that early environmental exposures shape long-term risk of CVDs, with particular emphasis on prenatal and early-life factors and their relationship to subsequent cardiovascular outcomes. Experimental and mechanistic studies were selectively included to support biological plausibility rather than to establish causality. Throughout this section, evidence from animal models, observational human studies, and interventional data is explicitly distinguished, and conclusions are framed in accordance with the strength and limitations of each study design.

### 5.1. Historical Famines and CVD Risk

Historical famines represent some of the earliest and best-studied natural experiments suggesting a link between prenatal undernutrition and long-term cardiovascular health. The Dutch Hunger Winter of 1944–1945 has been particularly informative. In a cohort study of individuals born during this period, those exposed to famine during early gestation exhibited a significantly higher prevalence of CHD (8.8%) compared with unexposed controls (3.2%), corresponding to an adjusted odds ratio of 3.0 after adjustment for sex. This association was independent of birth weight, suggesting that early gestational undernutrition may influence later cardiovascular risk through mechanisms extending beyond fetal growth restriction [92]. Subsequent analyses of the same cohort confirmed and expanded these findings, demonstrating that individuals conceived during the famine not only had a higher cumulative incidence of CAD (13%) but also developed the disease approximately three years earlier than those who were not exposed to famine [93]. Furthermore, an analysis of 7845 Dutch women from the Prospect-EPIC cohort showed that exposure to famine during adolescence (ages 10–17 years) in 1944–1945 was associated with a significantly increased risk of ischemic heart disease in adulthood, even after adjustment for socioeconomic and lifestyle factors. This observation extends the developmental origins of health hypothesis beyond the fetal period, suggesting that adolescence may represent an additional window of vulnerability to nutritional stress [94]. In contrast, a large cohort study including more than 41,000 Dutch individuals did not demonstrate an increase in cardiovascular mortality following prenatal famine exposure, although a modest increase in mortality from other causes was observed [95].

Consistent associations have also been observed in a large Chinese population affected by famine during the years 1959–1961. In a study including more than 259,000 adults, exposure to famine in early life was associated with a significantly increased risk of overall CVDs, MI, stroke, and coronary heart disease. The risk was highest among individuals from several of the most severely affected regions, supporting a dose–response relationship, but not establishing causality [96]. In the same context, data from the China Health and Retirement Longitudinal Survey indicated that exposure to famine during fetal development or early childhood was associated with a higher prevalence of CVDs in adulthood. The highest risk was observed among individuals who experienced famine during infancy, suggesting that infancy represents a particularly sensitive window for long-term health consequences [97]. Extending these observations beyond ischemic heart disease, a recent analysis from the China Patient-Centered Evaluative Assessment of Cardiac Events Million Persons Project demonstrated that individuals born during famine had a significantly increased risk of hospitalization for HF in adulthood compared with those born after the famine. This association remained significant after adjustment for confounding factors and was particularly pronounced among participants with hypertension, diabetes, or dyslipidemia [98]. Collectively, these observational human studies underscore the association between early-life undernutrition and later cardiovascular outcomes, while highlighting the inherent limitations of inferring causality from non-randomized human data.

### 5.2. Intergenerational and Transgenerational Influences

Intergenerational and transgenerational influences on cardiovascular health have been highlighted in studies examining historical variations in food availability. In Överkalix, Sweden, cohorts born in 1890, 1905, and 1920 were observed to assess whether exposure of parents or grandparents to famine or food abundance during the Slow Growth Period (SGP) affected the risk of CVDs and diabetes in descendants. The study demonstrated that limited food availability during the father’s SGP was associated with lower cardiovascular mortality in offspring. Associations observed between paternal exposure during the SGP and cardiovascular mortality in offspring primarily support an intergenerational model. In contrast, excessive nutrition during the paternal grandfather’s SGP increased the risk of diabetes which may be interpreted as suggestive of transgenerational associations [99]. A subsequent analysis of the 1905 cohort further explored these effects, hypothesizing a potential epigenetic mechanism involving genomic imprinting. In this study, excess food availability during the paternal grandfather’s SGP (at 9–12 years of age) was associated with reduced survival of descendants, while the influence of maternal parents or grandparents was negligible [100]. Additional evidence suggests that these effects may be sex-specific. Nutritional status of the paternal grandfather primarily influenced mortality risk in grandsons, whereas nutritional exposure of the paternal grandmother was associated with smoking behavior before 11 years of age and with growth patterns in children, compared with those whose fathers initiated smoking later. After adjustment for confounding factors, early prenatal exposure to smoking was associated with higher body mass index in sons at 9 years of age, but not in daughters [101]. These findings underscore that ancestral nutritional exposures may shape cardiometabolic risk in descendants not only through direct environmental effects but also via complex, sex-dependent intergenerational or transgenerational mechanisms.

### 5.3. Maternal Metabolic Disorders

Maternal metabolic disorders, such as diabetes and hypertensive disorders of pregnancy, have been consistently associated with adverse cardiovascular outcomes in offspring from early life through adulthood. Large population-based cohort studies from Denmark have demonstrated that children born to mothers with pregestational or gestational diabetes have a 29% higher risk of developing early-onset CVDs, including HF, hypertensive diseases, and thromboembolic events, up to 40 years of age. The risk was higher among offspring of mothers with diabetes-related complications or a history of CVDs [102]. Similarly, exposure to maternal hypertensive disorders of pregnancy, including preeclampsia, eclampsia, gestational hypertension, and preexisting hypertension, was associated with an increased risk of early-onset CVDs, with the highest risk observed in cases of severe or early-onset preeclampsia. Offspring of mothers with a combined history of CVDs or diabetes were particularly vulnerable [103]. These findings underscore that an adverse intrauterine metabolic environment can program the offspring’s cardiovascular system, highlighting the critical role of maternal health in shaping both early- and long-term CVD risk.

### 5.4. Advanced Maternal Age

Moreover, advanced maternal age is increasingly recognized as a factor potentially influencing the cardiovascular health of offspring. Experimental studies in animal models indicate that offspring born to older mothers are exposed to a less favorable intrauterine environment, leading to sex-dependent cardiovascular alterations in adulthood. In these models, males exhibit impaired endothelium-dependent relaxation, reduced endothelium-dependent hyperpolarization, and diminished recovery following ischemia–reperfusion, whereas females appear relatively protected due to greater reliance on NO-mediated mechanisms [104]. Further evidence from a controlled rat model confirms that advanced maternal age can induce sex-specific cardiac and vascular changes in offspring under experimental conditions. Offspring of older dams (9.5–10 months of age) exhibited sex-specific cardiac and vascular alterations in adulthood. At 12 months of age, only males showed signs of left ventricular diastolic dysfunction and impaired recovery after ischemia–reperfusion, whereas females demonstrated elevated SBP with preserved endothelial function [105]. While these animal experimental findings provide mechanistic insight, their direct applicability to human pregnancy and long-term cardiovascular risk remains uncertain and requires cautious interpretation.

### 5.5. Impact of Air Pollution

Exposure to air pollution is also increasingly recognized as an important determinant of cardiovascular health early in life. Experimental studies have shown that prenatal exposure of pregnant mice to concentrated particulate matter can result in lower birth weight and persistent cardiac dysfunction in adult offspring, including reduced ejection fraction, impaired contractility, and increased myocardial fibrosis. These findings suggest potential biological mechanisms, but cannot be directly extrapolated to humans [106]. Complementary evidence from observational human studies indicates similar effects. In a UK birth cohort followed from pregnancy to 18 years of age, higher cumulative exposure to PM_2.5_, NO_2_, and black carbon was associated with higher DBP and heart rate in early adulthood. Individuals with the highest lifetime exposure exhibited the most unfavorable cardiovascular profiles [107]. Complementary findings from the Hong Kong “Children of 1997” cohort demonstrated that early exposure to PM_10_ was associated with higher low-density lipoprotein concentrations in adolescent boys, particularly when exposure occurred in utero or during infancy, whereas no such associations were observed in girls [108]. Moreover, evidence from a large birth cohort conducted in Wuhan, China, suggests that maternal exposure to high concentrations of PM_2.5_ and PM_10_ early in pregnancy is associated with an increased risk of congenital heart defects, particularly ventricular septal defects. The risk increased monotonically with PM_2.5_ exposure during gestational weeks 7–10, indicating a critical window of developmental susceptibility of the heart to air pollution [109]. Together, these studies support a potential link between early-life air pollution exposure and cardiovascular vulnerability. However, human evidence remains observational, and animal data primarily inform biological plausibility rather than causality.

### 5.6. Effects of Tobacco Smoke Constituents

Exposure to tobacco smoke during pregnancy represents another key modifiable environmental factor associated with offspring cardiovascular health. Evidence from human studies indicates that maternal smoking during pregnancy may have both early and long-term cardiovascular consequences. In the Longitudinal Study of Australian Children, 11–12-year-olds with prenatal exposure to tobacco smoke exhibited a higher risk of hypertension compared with children of non-smoking mothers, with risk increasing according to the intensity and duration of exposure. However, offspring of mothers who quit smoking during pregnancy displayed cardiovascular profiles comparable to those of unexposed peers, supporting a potentially reversible component of risk [80]. Extending these findings into later life, a large cohort study from the UK Biobank including more than 414,000 participants demonstrated that perinatal exposure to maternal smoking was associated with a higher risk of CVDs, MI, and stroke in adulthood. Moreover, this risk was greater among individuals who themselves smoked in adulthood, indicating a synergistic effect of prenatal and later-life tobacco exposure [81]. Although no randomized evidence is available, the consistency of findings across large observational cohorts and exposure gradients strengthens confidence in the observed associations.

## 6. Discussion

**Figure 4:**
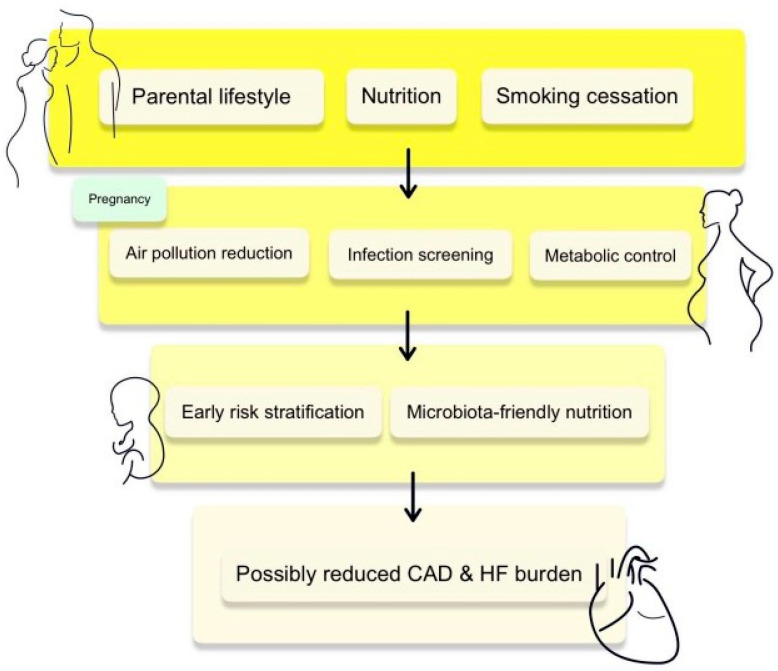
Preconception and early-life intervention strategies. Interventions across critical developmental windows potentially reduce long-term cardiovascular disease risk.

In recent decades, the mechanisms underlying epigenetic inheritance have been intensively investigated. Due to ethical constraints limiting such analyses in humans, most of the available evidence is derived from studies using animal models. Nevertheless, these studies leave substantial gaps, as no animal model to date has conclusively identified a mechanism capable of faithfully reproducing the intergenerational transmission of epigenetic traits observed in humans. Robust evidence from human population-based studies is also lacking, largely due to significant logistical, financial, and ethical barriers that hinder multigenerational research. An additional challenge is that many common environmental exposures affect individuals across multiple generations, which substantially complicates the distinction between truly inherited effects and those resulting from ongoing exposure [110,111]. Collectively, these limitations indicate that current evidence supports a cautious interpretation of epigenetic inheritance in cardiovascular disease, with stronger support for intergenerational influences than for true transgenerational transmission. The DOHaD concept emphasizes the critical role of material conditions in early life in shaping the risk of chronic disease and mortality. The prenatal period is particularly vulnerable, demonstrating high sensitivity to stressors such as undernutrition. According to the DOHaD framework, undernutrition during fetal life increases susceptibility to a wide range of conditions, including cardiovascular and cerebrovascular diseases and diabetes, and may also lead to impaired cognitive function and reduced socioeconomic attainment. Although this concept adopts a broad developmental perspective, most studies to date have focused primarily on prenatal exposures and cardiometabolic outcomes, thereby limiting a more comprehensive developmental approach. This narrowing of focus has constrained the integration of postnatal, adolescent, and intergenerational factors into a unified life-course model of cardiovascular risk. True transgenerational studies in human populations remain rare, largely due to ethical constraints associated with conducting randomized controlled trials. Most available analyses rely on observational data characterized by substantial methodological heterogeneity, varying exposure definitions, and difficulties in disentangling environmental effects from genetic factors [112,113]. Rather than acting through isolated biological pathways, early-life exposures appear to induce persistent biological reprogramming that affects vascular function, myocardial development, and systemic metabolic regulation, thereby contributing to shared downstream pathways leading to CAD and HF. Maternal and paternal exposure to traditional cardiovascular risk factors during critical time windows, including the preconception period or early pregnancy, may disrupt the epigenomic plasticity of the developing fetus. Consequently, maternal factors that modulate the overall risk of congenital heart defects in genetically predisposed offspring may represent promising targets for preventive interventions, highlighting the need for more in-depth research in this area [114,115,116]. In parallel, modifiable behavioral factors such as tobacco smoking, dietary patterns, physical activity levels, and obesity are well-established risk factors for CVDs. Emerging evidence further indicates that the presence of these risk factors in parents—including smoking, obesity, hypertension, and diabetes—not only affects their own disease risk but is also associated with an increased predisposition to CVDs in offspring, suggesting a transgenerational effect. In this context, epigenetic modifications—including chromatin remodeling, histone modifications, DNA methylation, regulation by non-coding RNAs, and epitranscriptomic changes—have emerged as key mediators linking behavioral and environmental factors with the development and progression of CVDs [17,82]. These epigenetic processes interact dynamically with mitochondrial function, inflammatory signaling, and metabolic pathways, forming interconnected regulatory networks rather than independent mechanisms. At the same time, it should be emphasized that no validated mechanism has yet been identified through which environmental factors could directly influence the epigenome. The hypothesis that certain forms of informational RNA may transmit a “memory” of environmental constraints via mammalian sperm has gained some support; however, it lacks robust experimental validation. In mammals, DNA methylation in gamete genomes and during early embryonic stages is largely reset, which limits its potential role as a carrier of intergenerational information [21]. This extensive epigenetic reprogramming represents a major biological barrier to the stable transmission of environmentally induced epigenetic marks across generations. Although available analyses are beginning to shed light on intergenerational mechanisms shaping cardiovascular health, the data remain limited by insufficient control for genetic factors. Future studies on the transgenerational transmission of CVD risk will benefit from the integration of molecular data from parents and their offspring, which may enable the construction of polygenic risk scores for specific components of CVDs. Moreover, studies capable of directly detecting environmental pollutants in tissues and linking them to clinical outcomes are needed. Such analyses will help determine whether exposure to pollutants should be classified as an independent cardiovascular risk factor or as an element that amplifies the effects of other well-established determinants, such as hypertension, diabetes, obesity, or tobacco smoking [71,117]. Addressing these methodological challenges will be essential for distinguishing causal biological programming effects from confounding by shared environmental and socioeconomic conditions. The effective translation of epigenetic research findings into clinical practice requires interdisciplinary collaboration, resolution of complex technical and ethical challenges, and the provision of adequate resources and expert knowledge. Epigenetics holds the potential to substantially transform approaches to cardiovascular and metabolic diseases by enabling the development of precision medicine and personalized healthcare, which may ultimately contribute to improved quality of life for millions of patients worldwide [118]. Despite efforts to comprehensively address the transgenerational impact on the risk of coronary artery disease and heart failure, our work acknowledges several methodological limitations. The diversity of tissue types, cellular heterogeneity, and lack of validation in independent cohorts may restrict result interpretation. Additionally, challenges in obtaining fetal tissues hinder high-quality analyses. Awareness of these limitations is crucial for accurate data evaluation and prioritizing future research directions. Figure 4 presents preconception and early-life cardiovascular intervention strategies.

## 7. Conclusions

Early exposure to environmental factors plays a critical role in the transgenerational development of CVDs. Research findings indicate that prevention should begin as early as the preconception period and include the promotion of healthy lifestyles among prospective parents. Key risk factors include air pollution, diet, smoking, and parental obesity, all of which significantly increase the likelihood of CVDs in offspring. This underscores the need to incorporate a transgenerational perspective, alongside intergenerational considerations, into public health strategies and to develop interventions that protect the health of both current and future generations. The evidence reviewed suggests that early-life and parental exposures influence cardiovascular risk through persistent biological reprogramming involving epigenetic regulation, metabolic pathways, inflammation, and vascular dysfunction. Furthermore, continued transgenerational and interdisciplinary research, supported by longitudinal human studies, may help elucidate epigenetic mechanisms and inform the design of effective preventive strategies.

## Figures and Tables

**Table 1:** Summary of key findings.

Mechanism	Key Changes	Cardiovascular Consequences	References
Epigenetic	DNA methylation, histone modifications (H3/H4, HAT/HDAC), alterations in miRNA expression (including inflamma-miRs), inheritance of successive DNA methylation patterns, modifications of promoters of metabolic genes (e.g., PPARα)	metabolic reprogramming, cardiomyocyte hypertrophy, mitochondrial dysfunction, ↑ ROS, ↑ susceptibility to CAD and HF	[30,31,32,33,34]
Mitochondrial	impaired mitochondrial biogenesis, dysregulation of oxidative phosphorylation, excessive production of ROS, prenatal hypoxia → persistent mitochondrial dysfunction	endothelial dysfunction, left ventricular hypertrophy, increased susceptibility to CVDs	[35,36,37]
Impact of Nutritional Deficiencies: Vitamin B12 and Folic Acid	disrupted methylation of RAA-related genes, altered NOS expression → ↓ NO, disrupted lipid and phospholipid homeostasis	endothelial dysfunction, hypertension, ↑ insulin resistance, greater susceptibility to CAD and HF	[38,39,40,41]
The Renin–Angiotensin–Aldosterone System and Histone Regulation	demethylation of the AGT promoter → ↑ angiotensinogen, ↑ Angiotensin I and Angiotensin II, histone modifications regulating inflammatory genes, regulation of HAT/HDAC affecting cardiomyocytes	↑ blood pressure, ↑ myocardial wall stress and hypertrophy, development of atherosclerosis	[42,43,44,45]
miRNA Role	altered miRNA profile in offspring, regulation of lipid metabolism, insulin sensitivity, and oxidative stress	chronic inflammation, endothelial dysfunction, ↑ risk of CAD	[46,47,48]
Role of the immune system	activation of NF-κB, ↑ IL-6, ↑ TNF-α, LPS exposure (prenatal infection model), chronic vascular inflammation	hypertension, accelerated atherosclerosis development, vascular dysfunction	[50,51]
Role of the gut microbiota	prenatal and infant dysbiosis, ↑ intestinal permeability, ↑ endotoxemia, activation of TLR4/MYD88/NF-κB, effects on lipid metabolism and body weight	chronic systemic inflammation, atherogenesis, metabolic disturbances promoting CAD and HF	[52,53,54,55,58]

Symbols used in the Table 1: ↑ increase; ↓ decrease; → leads to.

## Data Availability

The contributions presented in this study are included in the article. Further inquiries can be directed to the corresponding authors.

## Abbreviations

The following abbreviations are used in this manuscript:

WHO	World Health Organization
CVDs	Cardiovascular Diseases
DOHaD	Developmental Origins of Health and Disease
MI	Myocardial Infarction
HF	Heart Failure
CAD	Coronary Artery Disease
ROS	Reactive Oxygen Species
SBP	Systolic Blood Pressure
DBP	Diastolic Blood Pressure
SGP	Slow Growth Period
CHD	Congenital Heart Disease
NOS	Nitric Oxide Synthase
NO	Nitric Oxide
HDAC	Histone Deacetylase
HAT	Histone Acetyltransferases
TMAO	Trimethylamine N-oxide
